# Pyrazoles and Pyrazolines as Anti-Inflammatory Agents

**DOI:** 10.3390/molecules26113439

**Published:** 2021-06-05

**Authors:** Martha Mantzanidou, Eleni Pontiki, Dimitra Hadjipavlou-Litina

**Affiliations:** Department of Pharmaceutical Chemistry, School of Pharmacy, Faculty of Health Sciences, Aristotle University of Thessaloniki, 54124 Thessaloniki, Greece; martha19mantza@gmail.com (M.M.); epontiki@pharm.auth.gr (E.P.)

**Keywords:** pyrazolines, pyrazoles, antioxidant activities, anti-inflammatory activities, lipoxygenase inhibition, analgesic activity, anti-arthritis, docking study

## Abstract

The five-membered heterocyclic group of pyrazoles/pyrazolines plays important role in drug discovery. Pyrazoles and pyrazolines present a wide range of biological activities. The synthesis of the pyrazolines and pyrazole derivatives was accomplished *via* the condensation of the appropriate substituted aldehydes and acetophenones, suitable chalcones and hydrazine hydrate in absolute ethanol in the presence of drops of glacial acetic acid. The compounds are obtained in good yields 68–99% and their structure was confirmed using IR, ^1^H-NMR, ^13^C-NMR and elemental analysis. The novel derivatives were studied *in vitro* for their antioxidant, anti-lipid peroxidation (AAPH) activities and inhibitory activity of lipoxygenase. Both classes strongly inhibit lipid peroxidation. Compound **2g** was the most potent lipoxygenase inhibitor (IC_50_ = 80 µM). The inhibition of the carrageenin-induced paw edema (CPE) and nociception was also determined, with compounds **2d** and **2e** being the most potent. Compound **2e** inhibited nociception higher than **2d**. Pyrazoline **2d** was found to be active in a preliminary test, for the investigation of anti-adjuvant-induced disease (AID) activity. Pyrazoline derivatives were found to be more potent than pyrazoles. Docking studies of the most potent LOX inhibitor **2g** highlight hydrophobic interactions with VAL126, PHE143, VAL520 and LYS526 and a halogen bond between the chlorine atom and ARG182.

## 1. Introduction

Pyrazoles constitute a principal heterocyclic family containing two nitrogen atoms in their five-membered heterocyclic ring [1] exhibiting a wide range of chemical, biological, agrochemical and pharmacological properties [2]. Pyrazole is a versatile lead molecule; its derivatives are reported to possess innumerable biological activities such as anti-microbial, anti-fungal, anti-tubercular, anti-inflammatory, anti-convulsant, anticancer, anti-viral, angiotensin converting enzyme (ACE) inhibitory, neuroprotective, cholecystokinin-1 receptor antagonist, and estrogen receptor (ER) ligand activity [3]. Since 1883, when Knorr, L. [4] gave the generic name “pyrazole” to the above class of compounds synthesizing the first pyrazolin-5-one (3-methyl-1-phenyl-*2*-pyrazolin-*5*-one), many papers have reported the antipyretic, anti-inflammatory and analgesic activity of several pyrazoles, pyrazolin-3-ones and pyrazolidine-3,5-diones [5,6,7,8,9]. Many of these derivatives have been clinically applied as non- steroidal anti-inflammatory agents, such as anti-pyrine (2,3-dimethyl-1-phenyl-3-pyrazolin-5-one) or phenazone (analgesic and antipyretic), metamizole or dipyrone (analgesic and antipyretic), phenylbutazone (anti-inflammatory and antipyretic), aminopyrine or aminophenazone (anti-inflammatory, antipyretic and analgesic), sulfinpyrazone (chronic gout) and oxyphenbutazone (antipyretic, analgesic, anti-inflammatory, mild uricosuric) [10].

It is well known that non-steroidal anti-inflammatory drugs (NSAIDs) are important therapeutic agents for the treatment of various inflammatory disorders. The pharmacological activity is based on: (a) the suppression of prostaglandin biosynthesis from arachidonic acid *via* the inhibition of cyclooxygenases (COXs) and thromboxane synthase with a different degree of selectivity, and (b) the biotransformation of arachidonic acid *via* 5-lipoxygenase (5-LOX) to potent mediators of inflammation leukotrienes (LTs) and prostaglandins (PGs) [11,12,13]. COX enzymes exist in two isoforms: COX-1 (constitutive) expressed in most tissues and COX-2 (inducible) induced at sites of inflammation. Currently used NSAIDs exert their activity *via* the inhibition of both isoforms including major side effects at the gastrointestinal and renal level [14] due to their inhibition of COX-1-mediated physiological prostaglandins.

**Figure 1:**
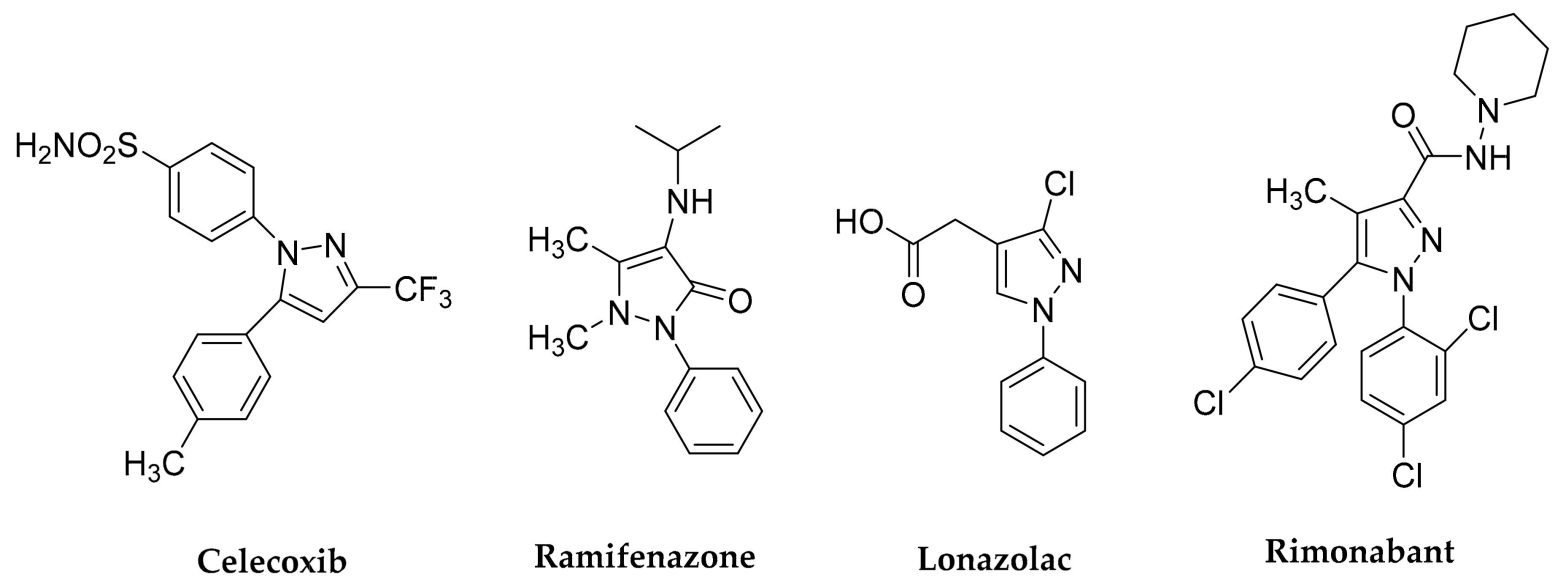
Structure of drugs bearing the pyrazole moiety.

Commercially available pyrazole moiety examples as potent COX-2 inhibitors are Celecoxib [15], Ramifenazone [16], Lonazolac (NSAID) [17] and Rimonabant [18] (Figure 1). The search for safer NSAIDs continues with the failure of anticipated “ideal” anti-inflammatory drugs, the coxibs, on long term usage [19]. Design and synthesis of NSAIDs with a potential for clinical use with less adverse side effects captured the heed of chemists and pharmacists.

Moreover, chalcones have played a crucial part in the development of heterocyclic compounds, and they have been used extensively in organic synthesis for the synthesis of several bioactive compounds. A classical synthesis of these compounds involves the base-catalyzed Claisen–Schmidt reaction of substituted ketones and aldehydes to give *α*, *β*-unsaturated ketones. Chalcones represent an important scaffold responsible for various biological activities such as anti-inflammatory, antimicrobial, antifungal, antioxidant and anticancer [20,21]. Thus, they can be used as intermediates undergoing a subsequent reaction with hydrazine hydrate affording pyrazoles/pyrazolines. It has been reported that pyrazolines possess analgesic, anti-inflammatory [22,23,24] and antimicrobial activities [25,26,27].

It is well known that, during inflammation, free radicals are produced, leading to peroxides and other reactive oxygen species [28]. Several researchers [29,30,31,32,33] have reported the implication of reactive oxygen species (ROS), e.g., hydroxyl radical, superoxide anion and hydrogen peroxide, in disorders associated with oxidative stress (e.g., coronary artery disease, inflammatory injury, cancer and cardiovascular diseases).

Based on these observations and in continuation with our work related to the synthesis of anti-inflammatory agents, we now describe the synthesis and the *in vitro* evaluation of a number of novel pyrazole and pyrazoline derivatives as antioxidants, lipoxygenase inhibitors and *in vivo* as anti-inflammatory and analgesic agents influencing adjuvant-induced arthritis.

## 2. Results and Discussion

### 2.1. Chemistry

**Scheme 1:**
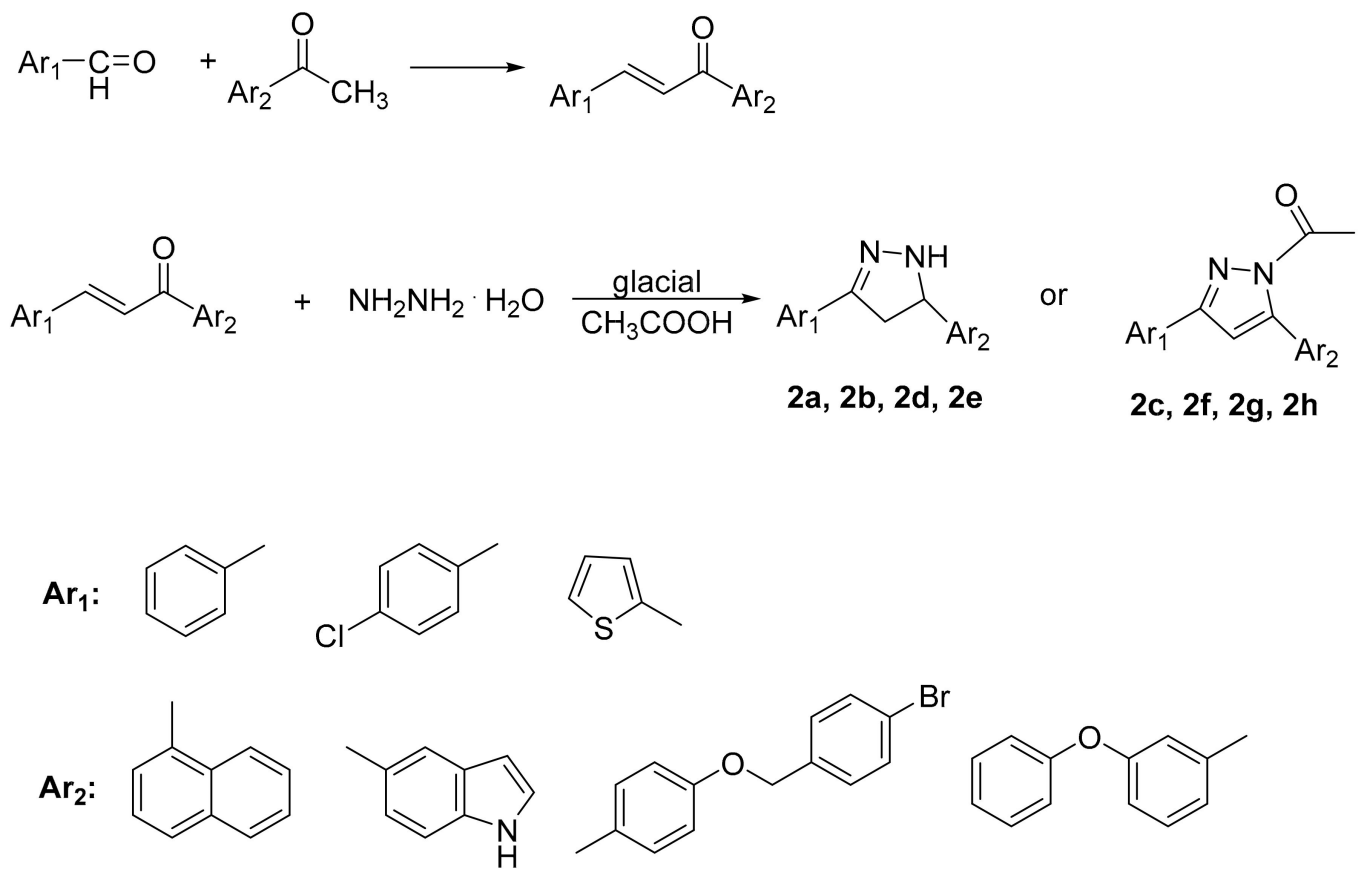
Synthesis of the novel derivatives.

The synthesis of the pyrazolines and pyrazole derivatives was accomplished *via* the condensation of the appropriate substituted aldehydes, suitable chalcones and hydrazine hydrate in absolute ethanol in the presence of drops of glacial acetic acid, as presented in Scheme 1 [34]. Chalcones as starting materials were successfully synthesized *via* Claisen–Italics Schmidt condensation using 15% KOH from the corresponding aldehydes with acetophenone in methanol (Scheme 1) [35].

Products **2a**–**2h** (Table 1) were obtained in satisfactory yields (68–99%). The pure final products were recrystallized from ethanol, acetone or preparative TLC while the chalcones were recrystallized from methanol. Structurally, compounds **2a**, **2b**, **2d** and **2e** are pyrazolines, whereas **2c**, **2f**, **2g**, **2h** are acetyl-substituted pyrazoles. Compound **2a** has been previously reported [36]. IR spectra for pyrazolines and pyrazole derivatives revealed the presence of a N-N bond at 1500–1510 cm^−1^, N-H at 3220–3400 cm^−1^ and C=N at 1660–1680 cm^−1^. ^1^H-NMR, ^13^C-NMR and elemental analysis were used for the confirmation of the synthesized compounds’ structures. The physical data of the synthesized compounds are given in detail in the Experimental Section.

### 2.2. Physicochemical Studies

#### 2.2.1. Determination of Lipophilicity

Lipophilicity is the key physicochemical parameter of a bioactive molecule, linking solubility, ligand-target binding interactions and membrane permeability with absorption, distribution, bioavailability, metabolism and elimination (ADME) and toxicological effects, crucial for its biological activity. The reverse phase thin layer chromatography (RPTLC) method was used for the experimental determination of the lipophilic character of the synthesized compounds as R_M_ values (Table 2) [37]. Lipophilicity was also theoretically calculated as clog *P* values, using the CLOGP Program of Biobyte Corp. [38] and as LPSP-lipophilicity values through Spartan v.5.1.3. (Wavefunction Inc., Irvine, CA, USA). According to the calculated clog *P* values, as well as LPSP, the most lipophilic compounds were the compounds **2c**, **2d**, **2e** and **2g**. This observation was supported by the R_M_ values (with the exception of **2g**).

Attempts to correlate clog *P* and R_M_ values as well as R_M_ values with LPSP values resulted to the following Equations (1) and (2).

clog *P* (C-QSAR) = 2.714 (±2.658) R_M_ + 4.515 (±1.474) (1)

n = 6, r = 0.817, r^2^ = 0.668, q^2^ = 0.222, s = 0.786, *F_1,4_* = 8.03, α = 0.05

R_M_ = 0.241 (±0.093) LPSP − 0.588 (±0.413) (2)

n = 7, r = 0.948, r^2^ = 0.898, q^2^ = 0.851, s = 0.117, *F_1,5_* = 43.84, α = 0.01

From our results (Table 2), it can be concluded that R_M_ values could be used as a successful relative measure of the overall lipophilic/hydrophilic properties of these molecules.

#### 2.2.2. Theoretical Calculation of Physicochemical Properties

The physicochemical properties were determined with the program Spartan v.5.1.3. (Wavefunction Inc., Irvine, CA, USA) in the conformation of minimum energy (Table 2).

### 2.3. Biological Evaluation

Reactive oxygen species and free radicals can be formed either from normal essential metabolic processes or external sources e.g., smoking, chemicals etc. [39]. They can be derived either from enzymatic (phagocytosis, prostaglandin synthesis, P-450) or non-enzymatic reactions (ionizing reactions, reaction of oxygen with organic compounds) [40]. Free radicals can be highly toxic, attacking macromolecules [41] leading to homeostatic disruption and cell damage, thus detoxification is of absolute necessity. Antioxidants (glutathione, ubiquinol, vitamin E, vitamin C etc) can delay or inhibit cellular damage due to their free radical scavenging activities and can terminate chain reactions before damaging vital molecules.

In this study, the novel derivatives were evaluated: (i) *in vitro* for their antioxidant activities and inhibition of soybean lipoxygenase, and (ii) *in vivo* for their anti-inflammatory activities using the carrageenin-induced edema, and for their analgesic activity, anti-nociception applying the writhing test and for the induction of adjuvant-induced disease (AID).

The antioxidant profile of the studied derivatives was determined through two different methods: (i) by measuring the scavenging ability by donating a hydrogen or an electron on a free radical, and (ii) by generating a free radical from an antioxidant system. The *in vitro* antioxidant activity was measured in terms of: (a) the interaction with the stable free radical DPPH; (b) the ABTS^+^ radical cation reduction-decolorization ability; and (c) anti-lipid peroxidation (AAPH). Factors such as solubility or steric hindrance seemed to be important, and influenced the experimental conditions.

The novel derivatives were studied for their interaction with the stable 2,2-diphenyl-1-picrylhydrazyl free radical (DPPH) at concentrations 50 µM, 100 µM, 200 µM and after 20 and 60 min (Table 3) [42]. This assay is based on the reduction of the DPPH by transferring an electron from the antioxidant. Nordihydroguaretic acid (NDGA) was used as a reference compound [43]. In general, the compounds present low or medium activity. However, it seems that the interaction is dependent on the concentration and on the reaction time. From this point of view, it can be concluded that the acetyl pyrazolyl derivatives **2c** and **2f** seem to present the highest scavenging activities. An increase in concentration favors the activity. On the contrary, time does not seem to affect the activity apart from compounds **2a** and **2d** at 100 µM. As for compounds **2a**, **2b**, **2c** and **2f**, activity seems to be time dependent at 200 µM. The acetyl pyrazole is more potent than the corresponding **2d** pyrazoline derivative. The presence of a chlorine group in **2e** pyrazoline leads to an activity increase (compared to **2d**) in concentration 200 µM. The acetyl derivative **2f** presents better activity than **2b** at 200 µM.

For the lipid peroxidation study, 2,2′-azobis(2-amidinopropane) hydrochloride (AAPH) was used for the generation of peroxyl radicals. The generation of the conjugated diene hydroperoxide derived from the oxidation of sodium linoleate in an aqueous solution was recorded at 234 nm [43,44]. The compounds presented high anti-lipid peroxidation activity (78–100%) (Table 4). Pyrazolines **2a** and **2b** present lower anti-lipid peroxidation activity (89% and 78% respectively). All the others are almost equally potent. Lipophilicity does not seem to influence this activity.

Pyrazolines **2d** and **2e** which are the most lipophilic compounds, showing antioxidant activity using the ABTS radical cation (ABTS^•+^) generated through potassium persulfate by oxidation with no participation of an intermediary radical. This reduction is completed by adding electron-donating antioxidants [43]. It seems that lipophilicity influences the activity, since both present high lipophilicity values. The compounds presented low to moderate antioxidant activity with most potent compound, **2e**, attributed to the presence of an electron acceptor substituent p-Cl at the molecule (Table 4).

Lipoxygenase (LOX) is the key enzyme implicated in membrane lipid peroxidation by forming hydroperoxides, thus it is considered a target for inflammatory diseases. LOX inhibitors may act either as radical scavengers or inhibitors of free radical production, since lipoxygenation occurs *via* a carbon centered radical [45]. LOX inhibitors bearing an antioxidant profile could be expected to offer protection in inflammatory conditions, and lead to potentially effective drugs. For the *in vitro* study, soybean lipoxygenase was used, based on the homology to mammalian lipoxygenase [46,47]. It has been found that, under the experimental conditions, all the synthesized derivatives inhibited soybean lipoxygenase (13–60%) apart from compound **2f**. Compound **2g** is the most potent among the synthesized derivatives. For the most promising compound, **2g**, IC_50_ value was calculated.

In an attempt to determine the type of LOX inhibition (competitive or non-competitive) for the most potent compound, **2g**, the study was conducted varying the concentration of the substrate, sodium linoleate (LLA) and keeping stable the enzyme’s and compound’s **2g** concentration. From the results, it can be concluded that **2g** LOX inhibition remained steadily strong, compared to the reference compound nordihydroguaeretic acid NDGA (Table 5). This underlines a competitive inhibition. In competitive inhibition, the inhibitor “competes” with the substrate at the binding site of the enzyme, and high substrate concentrations can break down the enzyme-inhibitor complex.

The carrageenin-induced rat paw edema assay was used, as a model of acute inflammation [48]. Inflammation induced by carrageenan is acute, non-immune, well-researched, and highly reproducible, and is described as a biphasic event. Cardinal signs of inflammation—edema, hyperalgesia, and erythema, are developed immediately following subcutaneous injection, resulting from the action of pro-inflammatory agents: bradykinin, histamine, tachykinins, complement reactive oxygen and nitrogen species. It is known that NSAIDs act during the second phase of prostaglandin release, presenting weak activity in the first phase of histamine and serotonin release.

Pyrazolines **2d** and **2e** presented the highest anti-inflammatory activity among the synthesized derivatives, exhibiting even higher activity compared to indomethacin used as a reference compound. Compounds were administered intraperitoneally in order to ensure systemic response *via* fast bioavailability, while carrageenin’s intradermal administration, as inflammatory agent, aimed at a local response. Compound **2a** was proven to be inactive and **2h** exhibited low activity. Compounds **2b**, **2c**, **2f** and **2g** presented medium activity. Lipophilicity seems to be important, since **2d** and **2e** are the most lipophilic derivatives within the set. The presence of a p-Cl at the benzyl ring slightly reduces the activity (**2e** < **2d**). It seems that the acetyl-substituted pyrazoles are less active.

Correlation of the *in vivo* anti-inflammatory activity (CPE%) with LPSP (lipophilicity values calculated from Spartan v.5.1.3), showed that lipophilicity governs the *in vivo* anti-inflammatory activity (Equation (3)).

log (CPE %) = 0.183 (±0.130) LPSP + 0.706 (±0.611)(3)

n = 6, r = 0.890, r^2^ = 0.793, q^2^ = 0.475, s = 0.111, *F_1,_*_4_ = 15.35, α = 0.05

Compounds **2d** and **2e**, presenting the highest anti-inflammatory activity, were examined for their analgesic activity as peripheral nociception using the writhing test. The acetic acid-induced writhing test is a quick, simple and reproducible method, despite the fact that it lacks specialization. Pyrazoline **2e** proved to be more effective than pyrazoline **2d** (Table 6).

Free radicals are particularly important in the inflammatory process [49,50,51]. ROS produced by phagocytes have been connected with the induction of inflammation and tissue damage. However, recently, ROS are also implicated in the regulation of inflammation and protection from autoimmunity. Evidence for the latter comes from association of ROS-deficiency with severe chronic inflammation in animal models and human patients in an ever-growing number of pathologic conditions, such as arthritis, lupus and neurodegenerative diseases.

Since it has been reported that anti-inflammatory drugs may also be effective in the prevention of free radical mediated damage [52], it is therefore to be considered that the action of some anti-inflammatory agents may be due to their antioxidant and free radical scavenging properties.

Taking into consideration the above, our biological findings as a whole show an agreement between anti-inflammatory and antioxidant activity for compounds **2c**, **2d**, **2e**, **2g**. They could indicate that organic peroxy-radicals, such as lipoperoxy-radicals, can be scavenged by these compounds, and this may be implicated in the mechanism of their anti-inflammatory ability. In the literature are referred, antioxidants possessing anti-inflammatory activity [52].

Compound **2d** exerts an effect greater than that of indomethacin, which has potent anti-inflammatory activity. The inhibition observed by compound **2d** was greater than that of indomethacin. Furthermore, the antioxidant-anti lipid peroxidation activities of compounds **2d** and **2e** are in correlation to their anti-inflammatory and analgesic activity.

Furthermore, it has been claimed that compounds acting as antioxidants could act as cyclooxygenase and/or lipoxygenase inhibitors [53].

Thus, the above tested compound **2d** appeared to be an effective agent not only on acute, but also on chronic inflammation of arthritis, and it is possibly effective in autoimmune diseases in correlation to its antioxidant activity. Rats treated with compound **2d** either did not develop or developed very mild arthritis, and simultaneously indicated anti-inflammatory activity.

**Figure 2:**
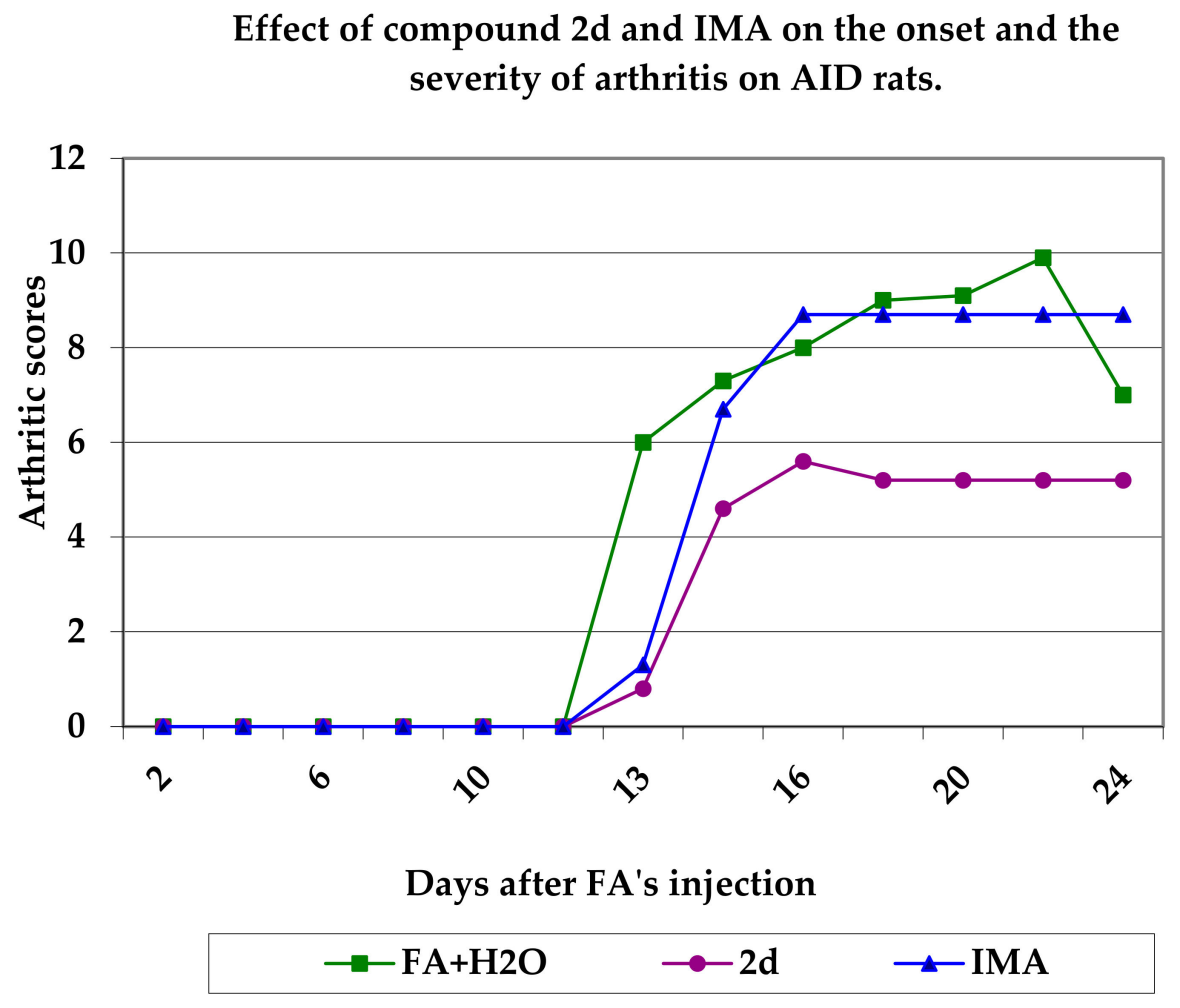
Effect of the compound **2d** and IMA on the onset and severity of arthritis in AID of rats.

Pyrazoline **2d** was selected, since it was found to be the most active of these compounds in a preliminary test, for the investigation of anti-adjuvant-induced disease (AID) activity. Adjuvant-induced disease (AID) is a good experimental model for rheumatoid arthritis, and is often used in testing agents for anti-inflammatory activity [54]. The compound was administered intraperitoneally on the 1st day, i.e., the day of the administration of Freund’s adjuvant (FA), and once every other day for the following 24 days. Adjuvant arthritis was developed about 14 days post-FA administration. Arthritic scores, body weight loss and *in vivo* zoxazolamine metabolism impairment (expressed as the duration of the induced paralysis) were recorded and found to be significantly reduced in comparison to the AID rat controls, which were treated only with the liquid vehicle. From these results it can be concluded that the examined pyrazolines possess preventive activity. The effect of the tested compound on inflammation is shown in Table 7 and the time course of adjuvant arthritis development expressed as arthritic score is shown in Figure 2. In the same experimental model, Indomethacin (IMA) was used as a reference compound.

### 2.4. Computational Studies–Docking Simulation Soybean Lipoxygenase

#### Molecular Modeling of the Synthesized Derivatives in Soybean LOX

*In silico* docking studies have been performed for all the synthesized derivatives, and their docking scores, hydrophobic interactions, hydrogen bonds, π-cation interactions and halogen bonds of the synthesized derivatives with different residues are given in Table S1 as Supplementary Material. Figure S1 presents the preferred docking poses of pyrazoles (**2a** [pink], **2b** [blue], **2d** [purple] and **2e** [cyan]) bound to soybean lipoxygenase (LOX-1) and Figure S2 presents the preferred docking poses of pyrazolines (**2c** [light sea green], **2f** [light purple], **2g** [light green] and **2h** [peach pink]) bound to soybean lipoxygenase (LOX-1) (Supplementary Material).

**Figure 3:**
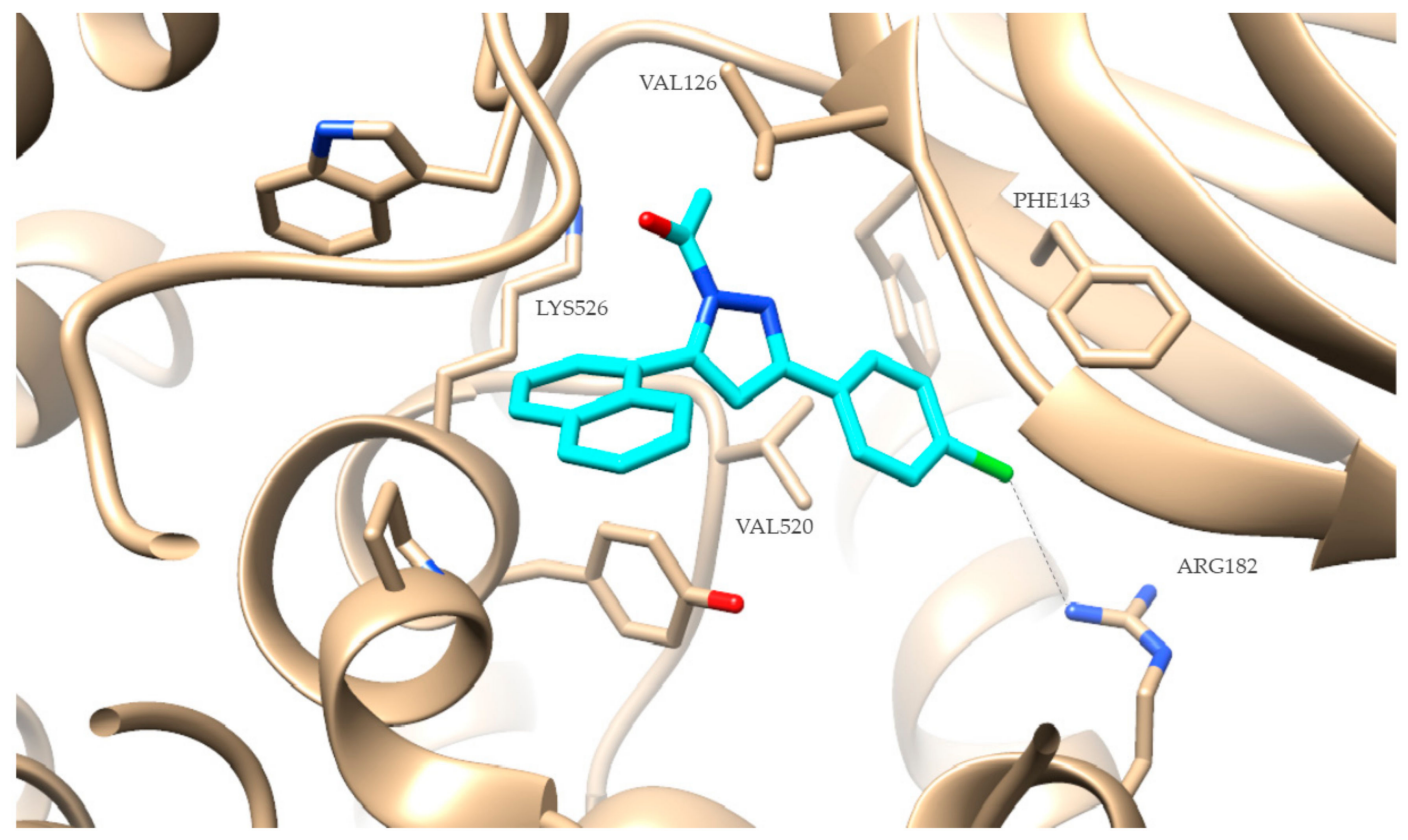
Preferred docking pose of **2g** (depicted in cyan) bound to soybean lipoxygenase (LOX-1).

The favored docking position of the most active derivative **2g** is shown in Figure 3. Compound **2g** has an AutoDockVina score of −10.3 kcal/mol binding to soybean LOX (PDB code: 3PZW). It is well known that one-to-one correlation is difficult to reach between the obtained results from the *in vitro* inhibition of soybean lipoxygenase that represent experimental values and docking scores that are based on algorithms and scoring function calculations. Docking describes the preferred orientation of the ligand bound to the protein. It seems that the novel compounds interact with the SLOX through allosteric interactions. Compound **2g** presents hydrophobic interactions with VAL126, PHE143, VAL520 and LYS526, and a halogen bond between the chlorine atom and ARG182. It is well known that most LOX inhibitors act as antioxidants or by scavenging free radicals [43], oxidizing the enzyme *via* a carbon-centered radical on a lipid chain. It is possible that compound **2g** exerts its activity by extending into the hydrophobic domain and blocking the substrates to the binding site, and thus preventing oxidation.

## 3. Experimental Section

### 3.1. Materials and Instruments

All chemicals, solvents, chemical and biochemical reagents were of analytical grade and purchased from commercial sources (Fluka, Alfa and Sigma-Aldrich, Merck). Soybean lipoxygenase, sodium linoleate, arachidonic acid (AA), 2,2-azobis (2-amidinopropane (ABTS), 2,2-azinobis-2-methyl-propanimidamine HCl (AAPH) were obtained from Sigma Chemical, Co. Nordihydroguaretic acid (NDGA), 1,1-diphenyl-2-picrylhydrazyl (DPPH), salycilic acid (SA), acetylsalicylic acid (ASA), 6-Hydroxy-2,5,7,8-tetramethyl-chroman-2-carboxylic acid (Trolox), Freund’s Adjuvant: Mycobacterium Butyricum (Difco-0640-33) were purchased from the Sigma-Aldrich Chemical Co. (St. Louis, MO, USA). All starting materials were obtained from commercial sources (Merck, Merck KGaA, Darmstadt, Germany, Fluka Sigma-Aldrich Laborchemikalien GmbH, Hannover, Germany, Alfa Aesar, Karlsruhe, Germany and Sigma, St. Louis, MO, USA) and used without further purification.

Melting points (uncorrected) were determined on a MEL-Temp II (Lab. Devices, Holliston, MA, USA). For the *in vitro* tests, UV-Vis spectra were obtained on a Perkin-Elmer 554 double beam spectrophotometer. Infrared spectra (film as Nujol mulls or KBr pellets) were recorded with Perkin-Elmer 597 spectrophotometer (Perkin-Elmer Corporation Ltd., Lane Beaconsfield, Bucks, UK).

The ^1^H Nucleic Magnetic Resonance (NMR) spectra were recorded at 300 MHz on a Bruker AM-300 spectrometer (Bruker AnalytischeMesstechnik GmbH, Rheinstetten, Germany) in CDCl_3_ or DMSO using tetramethylsilane as an internal standard unless otherwise stated. ^13^C-NMR spectra were obtained at 75.5 MHz on a Bruker AM-300 spectrometer in CDCl_3_ or DMSO solutions with tetramethylsilane as internal reference, unless otherwise stated. Chemical shifts are expressed in_(ppm) and coupling constants *J* in Hz. Mass spectra were determined on a LC-MS 2010 EV Shimadzu (Shimadzu, Kiyoto, Japan), using MeOH as solvent. Elemental analyses for C and H gave values acceptably close to the theoretical values (±0.4%) in a Perkin-Elmer 240B CHN analyzer (Perkin-Elmer Corporation Ltd., Lane Beaconsfield, Bucks, UK). Reactions were monitored by thin layer chromatography on 5554 F254 Silica gel/TLC cards (Merck and FlukaChemie GmbH Buchs, Steinheim, Switzerland). For preparative thin layer chromatography (prep TLC) Silica gel 60 F254, plates 2 mm, Merck KGaA ICH078057 were used.

### 3.2. Chemistry General Procedure

#### 3.2.1. Synthesis of Chalcones

A Claisen–Schmidt condensation was performed between acetophenone and the suitable substituted aryl aldehyde at a molar ratio of 1:1 in methanol [35]. Five milliliters (3 mL) of aqueous KOH (15%) was added and the mixture was stirred at room temperature for 24 h. Reaction completion was monitored by TLC. The pH was adjusted to acidic *via* the addition of three milliliters (3 mL) of CH_3_COOH. The mixture was cooled down with ice and the formed precipitate was filtered and washed with methanol.

All the synthesized chalcones have been already reported in the literature [35,55,56] except the following ones:

(*E*)-*3*-(*4*-((*4*-*bromobenzyl*)*oxy*)*phenyl*)-*1*-*phenylprop*-*2*-*en*-*1*-*one* (**1d**). Aurora Fine Chemicals LLCUSA, Austria. Yield: 50%; R_f_ = 0.8 (CH_2_Cl_2_); m.p. 45–47 °C; ΙR (Nujol) (cm^−1^): (*C=O*) 1680; ^1^H-NMR (CDCl_3_): δ, (ppm) 5.07 (2H, s), 6.97–8.02 (15H, m, aromatics and -CH=CH-); ^13^C-NMR (CDCl_3_): 69.4, 76.6, 77.4, 115.3, 120.2, 128.4, 128.4, 128.6, 129.0, 130.2, 131.8, 132.5, 135.5, 144.5, 160.5, 187.3; Anal. C, H, N. Calcd % (C_22_H_17_O_2_Br): C: 67.19, H: 4.36; Found % (C_22_H_17_O_2_Br): C: 66.79; H: 4.36.

(*E*)-*3*-(*4*-((*4*-*bromobenzyl*)*oxy*)*phenyl*)-*1*-(*4*-*chlorophenyl*)*prop*-*2*-*en*-*1*-*one* (**1e**). Yield: 100%; R_f_ = 0.95 (CH_2_Cl_2_); m.p. 175 °C; ΙR (Nujol) (cm^−1^): (*C=O*) 1700; ^1^H-NMR (CDCl_3_): δ, (ppm) 5.10 (2H, s), 6.97–7.96 (12H, m, aromatics and -CH=CH-); ^13^C-NMR (CDCl_3_): 40.0, 69.4, 76.6, 77.4, 115.3, 119.5, 122.1., 128.0, 128.9, 129.0, 129.8, 130.3, 131.8, 135.4, 145.0, 160.6, 189.2; Anal. C, H, N. Calcd% (C_22_H_16_O_2_BrCl): C: 61.78, H: 3.77; Found% (C_22_H_16_O_2_BrCl): C: 61.57; H: 3.86.

#### 3.2.2. Synthesis of Pyrazolines and Pyrazole Derivatives

In a round bottom flask, the appropriate substituted chalcone (0.01 mol) and hydrazine hydrate (99%, 0.02 mol) were dissolved in absolute ethanol while adding drops of glacial acetic acid. The solution was stirred and heated under reflux for 8–13 h. Reaction completion was monitored by TLC. The excess of the solvent was removed under reduced pressure and the remaining residue was treated with a mixture of ice/cold water. The solid/semi-solid residue was filtered. The pure final products were obtained *via* recrystallization from the suitable solvents, e.g., ethanol 95° or acetone or preparative TLC.

*5*-(*naphthalen*-*1*-*yl*)-*3*-*phenyl*-*4*,*5*-*dihydro*-*1H*-*pyrazole* (**2a**) [36]. Yield: 77%; R_f_ = 0.7 (CH_2_Cl_2_); m.p. 45–47 °C; ΙR (Nujol) (cm^−1^): 3346, 1688, 1600, 1560; UV (abs. C_2_H_5_OH): λ_max_ = 330, 280, 206, ε_max_ = 4600, 1100, 75,400; ^1^H-NMR (DMSO-d_6_, CDCl_3_): δ, (ppm) 2.07–2.16 (d, 1H, -NH-pyrazoline), 3.00–3.29 (dd, 1H, CH_2_), 3.66–3.74 (dd, 1H, CH_2_), 5.66–5.73 (t, 1H, −CH-pyrazoline), 7.34–8.27 (m, 12H, aromatics); ^13^C-NMR (DMSO-d_6_, CDCl_3_): 41.0, 47.1, 123.2, 123.5, 125.6, 126.0, 126.6, 127.2, 128.89, 128.96, 128.66, 129.15, 129.28, 130.2, 131.0, 133.5, 138.1, 152.1. Anal. C, H, N. Calcd % (C_19_H_15_N_2_): C: 84.10, H: 5.57; N: 10.32; Found% (C_19_H_15_N_2_): C: 84.45; H: 5.88, N: 10.20.

*5*-(*3*-(*4*-*chlorophenyl*)-*4*,*5*-*dihydro*-*1H*-*pyrazol*-*5*-*yl*)-*1H*-*indole* (**2b**). Yield: 99%; R_f_ = 0.5 (CH_2_Cl_2_); m.p. 96–98 °C; IR (Nujol) (cm^−1^): 3220–3470, 1680, 1520; UV (abs. C_2_H_5_OH): λ_max_ = 325, 255.5, ε_max_ = 5100, 13,760; ^1^H-NMR (DMSO-d_6_, CDCl_3_): δ, (ppm) 2.15–2.25 (d, 1H, NH pyrazoline), 3.06–3.14 (dd, 1H, CH_2_), 3.41–3.55 (dd, 1H, CH_2_), 5.00–5.06 (t, 1H, CH), 6.51–6.67 (m, 1H, -CH_β_ = indole), 6.79–6.88 (br, 1H, -CH_(6)_-indole), 7.16–7.87 (m, 7H, aromatics), 8.34 (s, 1H, -ΝH-indole); ^13^C-NMR (DMSO-d_6_, CDCl_3_): 41.8, 58.7, 102.9, 111.9, 118, 118.6, 125.3, 127.5, 129.0, 129.3, 130.2, 131.0, 133.0, 134.5, 135.0, 137.0, 151.0; Anal. C, H, N. Calcd % (C_17_H_13_N_3_Cl): C: 69.50, H: 4.45; N: 14.25; Found% (C_17_H_13_N_3_Cl): C: 69.46; H: 4.54, N: 14.39.

*1*-(*5*-(*4*-((*4*-*bromobenzyl*)*oxy*)*phenyl*)-*3*-(*thiophen*-*2*-*yl*)-*1H*-*pyrazol*-*1*-*yl*)*ethan*-*1*-*one* (**2c**). Yield: 68%; R_f_ = 0.8 (EtOH:PE, 1:2); m.p. 47–48 °C; IR (Nujol) (cm^−1^): 3150, 1720, 1510; UV (abs. C_2_H_5_OH): λ_max_ = 330, 270, 233.5, ε_max_ = 6600, 17,000, 15,100; ^1^H-NMR (DMSO-d_6_, CDCl_3_): δ, (ppm) 2.23–2.31 (m, 3H, -CH_3_), 5.04 (s, 2H, -OCH_2_-), 6.68 (s, 1H, -CH = pyrazole), 6.97–7.62 (m, 11H, aromatics); ^13^C-NMR (DMSO-d_6_, CDCl_3_): 2.7, 69.7, 103.0, 104.8, 114.0, 115.7, 123.0, 124.4, 125.0, 126.0, 127.3, 128.0, 129.0, 129.4, 130, 132, 132.2, 139.0, 140.0, 167.0; Anal. C, H, N. Calcd% (C_22_H_17_N_2_O_2_SBr): C: 58.28, H: 3.78; N: 6.18; Found % (C_22_H_17_N_2_O_2_SBr): C: 58.55; H: 4.18, N: 6.08.

*5*-(*4*-((*4*-*bromobenzyl*)*oxy*)*phenyl*)-*3*-*phenyl*-*4*,*5*-*dihydro*-*1H*-*pyrazole* (**2d**). Yield: 94%; R_f_ = 0.6 (CH_2_Cl_2_); m.p. 98–100 °C; IR (Nujol) (cm^−1^): 3360, 1660, 1517; UV (abs. C_2_H_5_OH): λ_max_ = 354, 262, 235, ε_max_ = 23,020, 16,800, 21,200; ^1^H-NMR (DMSO-d_6_, CDCl_3_): δ, (ppm) 2.06–2.09 (d, 1H, NH), 2.98–3.07 (dd, 1H, -CH-), 3.40–3.49 (dd, 1H, -CH-), 5.10 (s, 2H, -CH_2_-), 6.89–6.92 (d, 2H, aromatics), 7.26–7.55 (m, 11H, aromatics); ^13^C-NMR (DMSO-d_6_, CDCl_3_): 2.7, 69.7, 103.0, 104.8, 114.0, 115.7, 123.0, 124.4, 125.0, 126.0, 127.3, 128.0, 129.0, 129.4, 130.0, 132.0, 132.2, 139.0, 140.0, 167.0; Anal. C, H, N. Calcd% (C_22_H_19_N_2_OBr): C: 64.87, H: 4.70; N: 6.88; Found% (C_22_H_19_N_2_OBr): C: 64.53; H: 4.69, N: 6.82.

*5*-(*4*-((*4*-*bromobenzyl*)*oxy*)*phenyl*)-*3*-(*4*-*chlorophenyl*)-*4*,*5*-*dihydro*-*1H*-*pyrazole* (**2e**). Yield: 72%; R_f_ = 0.7 (CH_2_Cl_2_); m.p. 108–110 °C; IR (Nujol) (cm^−1^): 3320, 1660, 1510; UV (abs. C_2_H_5_OH): λ_max_ = 330, 263.5, 233, ε_max_ = 10,000, 21,560, 15,600; ^1^H-NMR (DMSO-d_6_, CDCl_3_): δ, (ppm) 2.06–2.09 (d, 1H, NH), 2.95–3.03 (dd, 1H, -CH_2_-pyrazoline), 3.37–3.46 (dd, 1H, CH_3_ pyrazoline), 4.85–4.92 (t, 1H, -CH-), 5.01 (s, 2H, -OCH_2_-), 6.90–6.93(d, 2H, aromatics), 7.26–7.60 (m, 10H, aromatics); ^13^C-NMR (DMSO-d_6_, CDCl_3_): 269.2, 99.0, 114.0, 114.8, 122.3, 127.0, 128.7, 128.9, 129.1, 129.3, 129.7, 130.2, 130.5, 130.7, 131.0, 131.6, 131.9, 135.0, 137.5, 149.8, 151.0, 165.6; Anal. C, H, N. Calcd% (C_22_H_17_N_2_OBrCl): C: 59.95, H: 3.89; N: 6.39; Found% (C_22_H_17_N_2_OBrCl): C: 60.02; H: 3.79, N: 6.03.

*1*-(*5*-(*1H*-*indol*-*5*-*yl*)-*3*-*phenyl*-*1H*-*pyrazol*-*1*-*yl*)*ethan*-*1*-*one* (**2f**). Yield: 83%; R_f_ = 0.8 (CH_2_Cl_2_); m.p. 65–66 °C; IR (Nujol) (cm^−1^): 3380–3200, 1720, 1500; UV (abs. C_2_H_5_OH): λ_max_ = 254, ε_max_ = 15,000; ^1^H-NMR (DMSO-d_6_, CDCl_3_): δ, (ppm) 2.39 (s, 3H, -CH_3_), 6.68 (s, 1H, –CH = pyrazole), 7.36–7.97 (m, 11H, aromatics and ΝH indole); ^13^C-NMR (DMSO-d_6_, CDCl_3_): 21.7, 102.0, 104.0, 111.9, 118.0, 119,.0 124.0, 126.0, 126.9, 127.6, 128.7, 129, 129.9, 133.2, 136.0, 144.0, 145.0, 147.0, 166.1; Anal. C, H, N. Calcd % (C_19_H_15_N_3_O): C: 75.73, H: 5.02; N: 13.94; Found % (C_19_H_15_N_3_O): C: 75.89; H: 5.40, N: 13.80.

*1*-(*3*-(*4*-*chlorophenyl*)-*5*-(*naphthalen*-*1*-*yl*)-*1H*-*pyrazol*-*1*-*yl*)*ethan*-*1*-*one* (**2g**). Yield: 94%; R_f_ = 0.8 (CH_2_Cl_2_); m.p. 53–55 °C; IR (Nujol) (cm^−1^): 3100, 1680, 1560; UV (abs. C_2_H_5_OH): λ_max_ = 280, 247.5, ε_max_ = 11,600, 14,340; ^1^H-NMR (DMSO-d_6_, CDCl_3_): δ, (ppm) 2.59 (s, 3H, -CH_3_), 6.80 (s, 1H, -CH = pyrazole), 7.41–8.02 (m, 11H, aromatics); ^13^C-NMR (DMSO-d_6_, CDCl_3_): 23.0, 104.0, 124.4, 125.9, 126.7, 127.3, 127.7, 128.3, 128.9, 129.3, 129.8, 130.0, 130.8, 131.0, 131.8, 132.0, 134.0, 143.0, 145.0, 147.0, 169.8; Anal. C, H, N. Calcd % (C_21_H_15_N_2_OCl): C: 72.73, H: 4.36; N: 8.08; Found % (C_21_H_15_N_2_OCl): C: 72.35; H: 4.45, N: 8.12.

*1*-(*5*-(*3*-*phenoxyphenyl*)-*3*-*phenyl*-*1H*-*pyrazol*-*1*-*yl*)*ethan*-*1*-*one* (**2h**). Yield: 71%; R_f_ = 0.7 (CH_2_Cl_2_); m.p. 44–46 °C; ΙR (Nujol) (cm^−1^): 3100, 1718, 1680, 1500; UV (abs. C_2_H_5_OH): λ_max_ = 241, ε_max_ = 26,180; ^1^H-NMR (DMSO-d_6_, CDCl_3_): δ, (ppm) 2.78 (s, 3H, -CH_3_), 6.82 (s, 1H, –CH = pyrazole), 6.99–7.15 (m, 3H, aromatics), 7.33–7.71 (m, 11H, aromatics); ^13^C-NMR (DMSO-d_6_, CDCl_3_): 29.0, 100.4, 110.2, 116.0, 118.6, 119.0, 120.4, 123.5, 125.5, 128.5, 128.8, 129.8, 130.3, 140.4, 157.8, 176.9; Anal. C, H, N. Calcd% (C_23_H_18_N_2_O_2_): C: 77.95, H: 5.12; N: 7.90; Found% (C_23_H_18_N_2_O_2_): C: 78.05; H: 5.28, N: 8.28.

### 3.3. Physicochemical Studies

#### Experimental Determination of Lipophilicity as R_M_ Values

Lipophilicity as R_M_ values was determined through reversed phase TLC (RP-TLC) on silica gel plates impregnated with 5% (*v*/*v*) liquid paraffin in light petroleum ether. Methanol/water mixture (70/30, *v*/*v*) was used a mobile phase. Closed chromatography tanks saturated with the mobile phase at 24 °C were used for the development of the plates while the spots were detected under UV light. For the determination of R_M_ values, five individual measurements of R_f_ values were recorded, and the R_M_ values were derived from the equation R_M_ = log [(1/R_f_) − 1] [42,43] (Table 2).

### 3.4. Biological Assays

#### 3.4.1. Biological *In Vitro* Assays

A stock solution (1% DMSO in the appropriate buffer with the tested compound diluted under sonication) was prepared for the *in vitro* assays from which several dilutions were made with the appropriate buffer. The *in vitro* experiments were performed at least in triplicate, and the standard deviation of absorbance was less than 10% of the mean.

##### Determination of the Reducing Activity of the Stable Radical 1,1-Diphenyl-Picrylhydrazyl (DPPH)

A stock solution (10 mM) of the novel derivatives in DMSO was prepared and an equal volume of it was added to a solution of DPPH in absolute ethanol to reach final concentrations 50, 100 µM and 200 µM. The absorbance was recorded at 517 nm, after 20 and 60 min at room temperature (Table 3) [42,43].

##### Inhibition of Linoleic Acid Lipid Peroxidation

AAPH was used as free radical initiator of alkylperoxyl radicals. The ability of the compounds to prevent the oxidation of linoleic acid sodium salt from alkylperoxyl radicals was recorded at 234 nm. Trolox was used as reference (93%) (Table 4) [43,44].

##### ABTS+ -Decolorization Assay for Antioxidant Activity

The ABTS radical cation (ABTS^+•^) was generated by mixing ABTS stock solution in water (7 mM) with potassium persulfate (2.45 mM) and left in the dark at room temperature for 12–16 h before use. The results are recorded immediately after the mixing solutions at 734 nm. The results were compared to the appropriate standard inhibitor Trolox (Table 4) [44].

##### Soybean Lipoxygenase Inhibition Study

The stock solutions of the compounds were incubated with sodium linoleate (0.1 mM) and 0.2 mL of soybean lipoxygenase solution (1/9 × 10^−4^ *w*/*v* in saline) at room temperature. *In vitro* test was performed as previously published [42,43,44]. The test follows our previously published methods [42,43,44] The production of 13-hydroperoxylinoleic acid was recorded at 234 nm using using NDGA as reference compound (Table 3, Table 5).

#### 3.4.2. Biological *In Vivo* Assays

The animals (Fisher 344 rats), were housed under standard conditions, and received a diet of commercial food pellets and water ad libitum during the maintenance, but were entirely fasted during the experiment period. Both sexes were used, while pregnant females were excluded. Each group was composed of 6–15 animals. Our studies were in accordance with recognized guidelines on animal experimentation (guidelines for the care and use of laboratory animals published by the Greek Government 160/1991, based on EU regulations 86/609). Rats were kept in the Centre of the School of Veterinary Medicine (EL54 BIO42), Aristotle University of Thessaloniki, which is registered by the official state veterinary authorities (presidential degree 56/2013, in harmony with the European Directive 2010/63/EEC). The experimental protocols were approved by the Animal Ethics Committee of the Prefecture of Central Macedonia.

##### Inhibition of the Carrageenin-Induced Edema

Carrageenin in water as an intradermal injection of 0.1 mL 2% was used for the induction of the edema in the right hind paw of the rats, according to the previously reported method [44]. Animals were divided in five-membered groups and the tested compounds were suspended in water (0.0057 mmol/kg body weight), with a few drops of Tween 80, ground in a mortar before administered intraperitoneally simultaneously. The rats were euthanized 3.5 h after carrageenin injection. Comparison of the change in paw weight with that in control animals (treated with water) was expressed as a percent inhibition of the oedema CPE% values using indomethacin (IMA) as a reference compound. Values CPE% are the mean from two different experiments with a standard error of the mean less than 10% (Table 5). Student’s T test was applied.

##### Anti-Nociception-Writhing Test

For the study of the analgesic activity of the tested compounds, pain is provoked with acetic acid. Animals were divided in three five-membered groups, according to our reported method [44]. After treatment with the tested compounds (0.0057 mmol/kg body weight; intraperitoneally) in the first group, the second group was used as a positive control (aspirin; 0.0057 mmol/kg body weight i.p.) and the third group served as the control in which saline was administered (negative control). After 30 min, 0.6% acetic acid (1 mL/kg body weight) was injected i.p. The number of writhes was recorded for each animal every 5 min during a subsequent 30 min period (Table 6).

##### Induction of Adjuvant-Induced Disease (AID)

Rats were divided into four groups (five-membered groups of animals were used): groups one, two and three were injected intradermally [4] with 0.1 mL Freund’s adjuvant (FA) into the base of the tails of the animals; group three was the FA control rats; group four was injected with saline, used as an absolute control. Compound **2d** (group 1) and indomethacin (group 2) were injected i.p. once every other day for the following 24 days in a dose of 0.00057 mmol/mL/kg body weight (Table 7). Adjuvant arthritis was developed circa 14 days post-FA administration. Arthritic score was measured every 2 days from the commencement (14th day) day. Arthritic score was assessed on the 24th day. For quantification of arthritis (arthritic score); a single point was assigned for each wrist or ankle area and an additional point was given for each involved phalangeal joint, up to a maximum of five points per extremity [54,57,58].

On the 24th day and at least 12 h after the last injection, animals were administered zoxazolamine i.p. 10 mg/1 mL/g, as an aqueous suspension with a few drops of Tween 80, and the duration of the paralysis were assessed (Table 7) (Figure 2). The experiment was conducted in duplicate.

### 3.5. Computational Methods. Molecular Docking Studies on Soybean Lipoxygenase

For the docking studies, soybean lipoxygenase (PDB code: 3PZW) was used, and the visualization was accomplished through UCSF Chimera [59]. The protein was prepared: water molecules were removed, missing residues were added with Modeller [60], hydrogen atoms and AMBER99SB-ILDN charges were added, and the charge on iron was set to +2.0, with no restraint applied to the iron atom and the ligands. OpenBabel was used to generate and minimize ligand 3D coordinates using the MMFF94 force field [61]. Ligand topologies and parameters were generated by ACPYPE (AnteChamberPYthon Parser interfacE) [62] using Antechamber [63]. Energy minimizations were carried out using the AMBER99SB-ILDN force field [64] with GROMACS 4.6. Docking was performed with AutoDockVina (1.1.2) [65] applying a grid box of size 100 Å, 70 Å, 70 Å in X, Y, Z dimensions. The generation of docking input files and the analysis of the docking results was accomplished by UCSF-Chimera. Docking was carried out with an exhaustiveness value of 10 and a maximum output of 20 docking modes.

## 4. Conclusions

The synthesized compounds present antioxidant and anti-inflammatory activities, scavenging of free radicals, and inhibition of lipid peroxidation.

In the DPPH assay, the novel derivatives showed medium antioxidant activity with small differences dependent on time and concentration. Only compounds **2d** and **2e** moderately reduced the ABTS radical cation (ABTS^•+^) at 100 μM, while all compounds highly inhibited lipid peroxidation. Lipophilicity plays a significant role.

Compound **2g** presents the best lipoxygenase inhibition within the data test, in a competitive mode. Docking studies reveal that **2g** possibly interacts in an allosteric mode, presenting hydrophobic interactions with VAL126, PHE143, VAL520 and LYS526 and a halogen bond between the chlorine atom and ARG182.

Pyrazolines **2d** and **2e** seem to be the best anti-inflammatory agents. Simultaneously, they present satisfactory analgesic activity, whereas **2d** diminishes the severity and the onset of adjuvant-induced arthritis. Thus, it can be considered to be a lead compound with a multifunctional profile.

## Figures and Tables

**Table 1:** Substituted pyrazoles and pyrazoline derivatives.

Compd	Template	Ar_1_	Ar_2_
**2a**	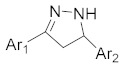	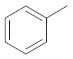	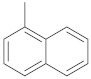
**2b**		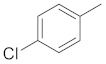	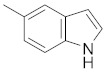
**2c**		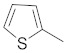	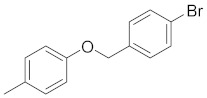
**2d**		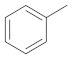	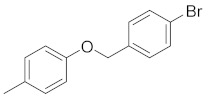
**2e**	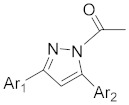	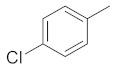	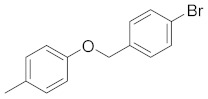
**2f**		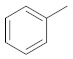	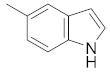
**2g**		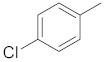	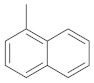
**2h**		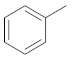	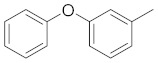

**Table 2:** Theoretical calculation of the properties associated with energy and charge distribution with the program Spartan v.5.1.3. Lipophilicity values: experimental R_M_% (R_M_ values are the average of at least five measurements; SD: standard deviation < 10%). Theoretically calculated clog *P* values calculated using the C-QSAR Program, Biobyte.

Compd.	*E*_(HOMO)_ (eV)	*E*_(LUMO)_ (eV)	Δ*E*_(HOMO-LUMO)_ (eV)	SM_2_ (kcal/mol)	Surface (Ǻ^2^)	(Ǻ^3^) (MV)	Dipole (D)	LPSP	R_M_^a^ (±SD) ^a^	clog *P* (C-QSAR)
**2a**	−7.65	2.15	9.80	−6.29	321.20	318.74	2.66	4.19	0.22 ± 0.2	4.77
**2b**	−7.67	2.64	10.31	−11.04	329.46	320.31	5.84	3.36	0.23 ± 0.03	4.30
**2c**	−8.44	2.39	10.83	−25.72	450.99	439.09	2.44	4.84	0.68 ± 0.05	7.40
**2d**	−7.84	2.86	10.70	−8.24	419.96	410.67	1.57	5.63	0.72 ± 0.06	6.15
**2e**	−8.08	2.78	10.86	−10.96	434.69	422.90	4.27	5.96	0.87 ± 0.07	6.69
**2f**	−7.73	2.48	10.21	−12.79	349.38	340.28	2.32	2.22	−0.07 ± 0.01	4.97
**2g**	−7.78	2.07	9.85	−7.37	382.80	376.11	1.44	4.17	−0.59 ± 0.05	6.89
**2h**	−8.46	2.23	10.69	−7.27	412.08	403.67	2.38	3.64	0.41 ± 0.02	6.78

^a^ SD standard deviation < 10%.

**Table 3:** Interaction with the stable radical 1,1-diphenyl-picrylhydrazyl (DPPH), *In vitro* lipoxygenase (LOX) inhibitory activity at 100 µM (LOX%).

Compd.	RA% 50 µM 20 min	RA% 50 µM 60 min	RA% 100 µM 20 min	RA% 100 µM 60 min	RA% 200 µM 20 min	RA% 200 µM 60 min	LOX % Inhibition at 100 µM
**2a**	15.0	20.0	22.9	32.0	37.2	49.3	35
**2b**	19.3	25.0	26.1	33.6	34.9	44.4	17
**2c**	17.1	22.4	41.9	44.0	49.4	59.2	42
**2d**	6.3	9.5	19.4	37.4	9.4	14.9	13
**2e**	9.5	13.0	14.8	17.8	25.6	30.1	16
**2f**	21.0	24.7	34.7	38.8	50.1	59.5	3
**2g**	9.0	12.7	11.2	13.6	22.5	27.7	60 (IC_50_ = 80 μM)
**2h**	6.7	9.5	7.8	9.6	17.2	20.9	26
**NDGA**	81	83	87	93	94	96	93 (0.45 μΜ)

**Table 4:** % Anti-lipid peroxidation (AAPH), decolorization activity ABTS^+^ % assays. *In vivo* anti-inflammatory activity (CPE%).

Compd.	AAPH% 100 µM	ABTS^+^ % 100 µM	CPE ^a^ %
**2a**	89	no	no
**2b**	78	no	27.0 *
**2c**	100	no	38.0 *
**2d**	96	15	63.0 **
**2e**	98	30	56.0 **
**2f**	95	no	30.0 *
**2g**	100	no	33.0 *
**2h**	97	no	16.0 *
**Trolox**	93	91	-
**Indomethacin**	-	-	47 **

* *p* < 0.01, ** *p* < 0.05. ^a^ % of reduction in the rat paw edema (CPE%) induced by carrageenin at the dose of 0.0057 mmol/Kg/body weight. No: no action under the experimental conditions.

**Table 5:** *In vitro* assay for the determination of the type of inhibition of lipoxygenase (LOX).

LLA *-C	2g-LOX (% Inhibition 100 µM)	NDGA-LOX (% Inhibition 100 µM)
50 µM	89.6	39.3
100 µM	-	36.2
200 µM	-	6.6

* Linoleic acid sodium salt concentration.

**Table 6:** *In vivo* analgesic activities of **2d** and **2e**, % inhibition of writhing responses (Writhing inhibition%).

Compound	Writhing Inhibition (%) ^a^
2d	54.2 *
2e	66.1 *
Aspirin	77 **

* *p* < 0.01, ** *p* < 0.05; ^a^ Dose of the administered 0.0057 mmol/kg body weight.

**Table 7:** Assessment of the preventive action of **2d** on the adjuvant-induced disease (AID). Manifestations (body weight change, liver weight change, zoxazolamine paralysis) in comparison to indomethacin (IMA), used as a reference compound.

Examined Parameters (mean ± SD)	AID Rats Treated with 2d	AID Rats Treated with IMA	AID Rats-Controls Treated only with the Liquid Vehicle	Absolute Controls, Normal Animals Treated only with the Liquid Vehicle
Percent change in body weight (g ± SD)	6 ± 0.3 **	8.3 ± 0.2 *	3 ± 0.1 *	14 ± 2 *
Percent change in liver weight (g ± SD)	7 ± 0.6 *	6.3 ± 0.4 *	7.8 ± 0.4 *	8.7 ± 0.5 **
Zoxazolamine paralysis (minutes ± SD)	227 ± 17 *	217 ± 17 **	284 ± 19 *	156 ± 18 *

* *p* < 0.01 (Student’s Test), ** *p* < 0.005 (Student’s Test).

## Data Availability

All the data of this research is included in this paper.

## Supplementary Materials

Table S1: Docking scores. Hydrophobic interactions, hydrogen bonds, π-cation interactions and halogen bonds of the synthesized derivatives with different residues, Figure S1: Preferred docking poses of pyrazoles (**2a** [pink], **2b** [blue], **2d** [purple] and **2e** [cyan]) bound to soybean lipoxygenase (LOX-1), Figure S2: Preferred docking poses of pyrazolines (**2c** [light sea green], **2f** [light purple], **2g** [light green] and **2h** [peach pink]) bound to soybean lipoxygenase (LOX-1).

## Sample Availability

Samples of the compounds (**2a**–**2h**) are available from the authors.

## Abbreviations

AA: Arachidonic acid; AAPH: 2,2′-azinobis(2-amidinopropane) hydrochloride; ACE: angiotensin converting enzyme; ADME: Absorption, Distribution, Bioavailability, Metabolism and Elimination; AID: Adjuvant-induced disease; clog *P*: theoretical calculated lipophilicity; COX: cycloxygenase; CPE: carrageenin-induced rat paw edema; DPPH: 2,2-diphenyl-1-picrylhydrazyl free radical; ER: estrogen receptor; LOX: Lipoxygenase; LPSP: lipophilicity values through Spartan v.5.1.3.; LTs: leucotrienes; NDGA: nordihydroguaeretic acid; NSAIDs: non-steroidal anti-inflammatory drugs; PGs: prostaglandins; QSAR: Quantitative Structure Activity Relationships; ROS: Reactive Oxygen Species; RPTLC: Reverse Phase Thin Layer Chromatography.
