# Supplementary material for: Pyrazoles and Pyrazolines as Anti-Inflammatory Agents
Source: Molecules. 2021 Jun 5;26(11):3439. doi: 10.3390/molecules26113439 (PMC8201324; doi:10.3390/molecules26113439)
Supplement: Supplementary file 1 [file molecules-26-03439-s001.zip › molecules-1235665-supplementary.pdf]

Supplementary Information

Computational Studies – Docking Simulation Soybean Lipoxygenase

Molecular Modeling of the synthesized derivatives in soybean LOX

Table S1. Docking scores. Hydrophobic interactions, hydrogen bonds, π-cation interactions and halogen bonds of the synthesized derivatives with different residues

compd	Docking Scores (kcal/mol)	Hydrophobic interactions	Hydrogen bonds	π-cation interactions	Halogen bonds
**2a**	-9.6	VAL126, VAL520, TYR525, PRO530, ARG533, VAL762, ASP768, TRP772	THR529	-	-
**2b**	-9.4	VAL126, VAL520, TYR525, PRO530, ASP768	LYS526, THR529, ARG767	LYS526	PHE144
**2c**	-9.6	LEU169, TYR184, ASP243, VAL520, ARG533, ASP768	TYR184	-	-
**2d**	-10.3	PHE143, LEU169, VAL520, LYS526, PRO530, TRP772		ARG182	TYR184
**2e**	-10.1	VAL126, LEU246, VAL520, ARG533, TRP772	ASP768		GLU244
**2f**	-10.1	VAL126, PHE143, VAL520	ASP768		
**2g**	-10.3	VAL126, PHE143, VAL520, LYS526	-	-	ARG182
**2h**	-10.0	VAL126, PHE143, VAL520	-	-	-

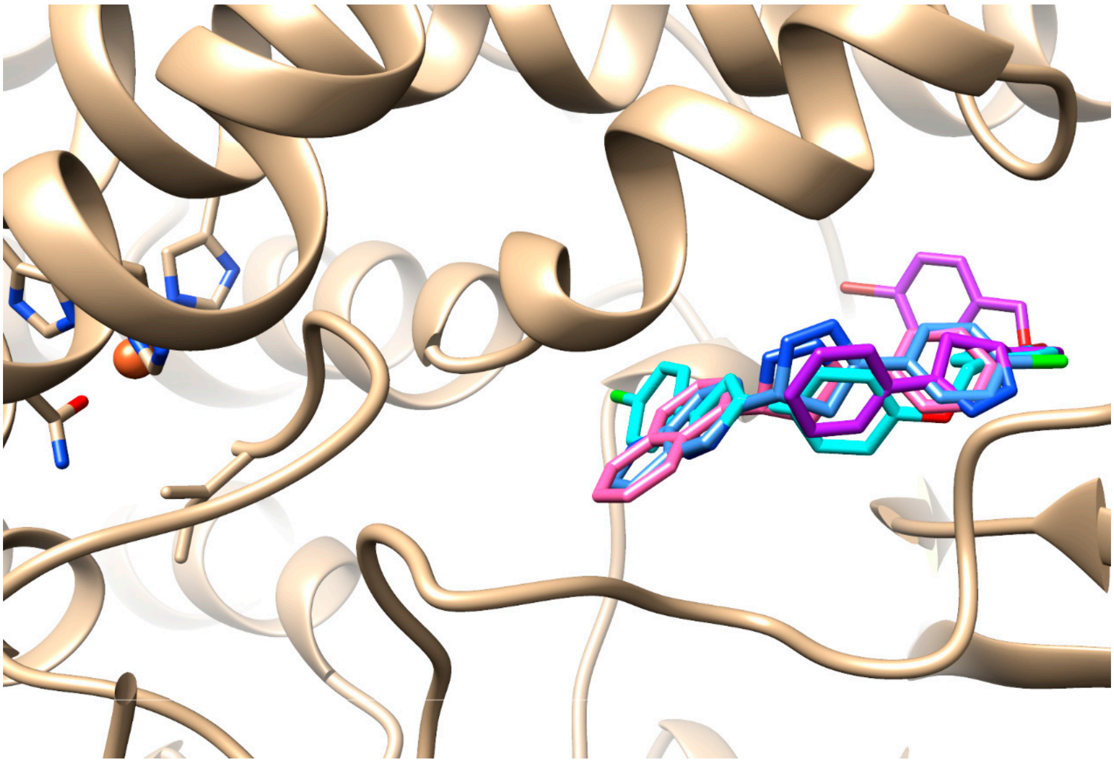

Figure S1. Preferred docking poses of pyrazoles (**2a** [pink], **2b** [blue], **2d** [purple] and **2e** [cyan]) bound to soybean lipoxygenase (LOX-1).

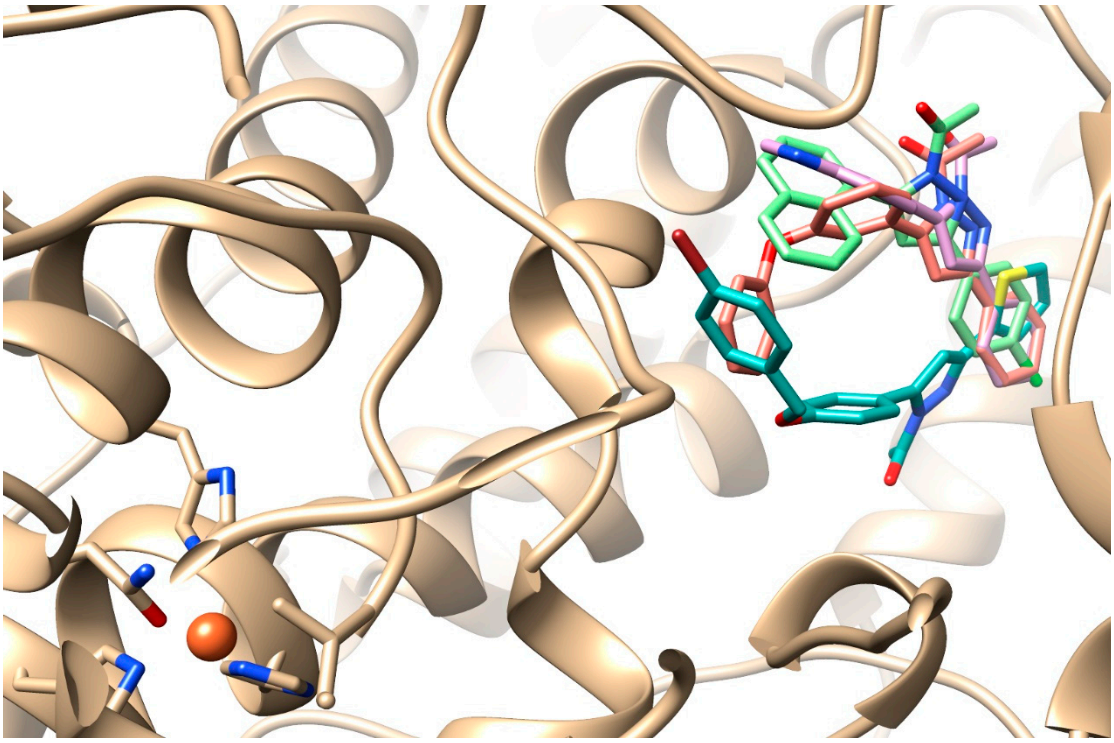

Figure S2. Preferred docking poses of pyrazolines (**2c** [light sea green], **2f** [light purple], **2g** [light green] and **2h** [peach pink]) bound to soybean lipoxygenase (LOX-1).
